# New insight into ischemic stroke: Circadian rhythm in post-stroke angiogenesis

**DOI:** 10.3389/fphar.2022.927506

**Published:** 2022-08-09

**Authors:** Yuxing Zhang, Lijuan Liu, Xin Zhao, Siyang Yan, Fukang Zeng, Desheng Zhou

**Affiliations:** ^1^ The Graduate School, Hunan University of Chinese Medicine, Changsha, Hunan, China; ^2^ Key Laboratory of Hunan Province for Integrated Traditional Chinese and Western Medicine on Prevention and Treatment of Cardio-Cerebral Diseases, Changsha, China; ^3^ Department of Neurology, The First Affiliated Hospital of Hunan University of Chinese Medicine, Changsha, Hunan, China; ^4^ The Medical School, Hunan University of Chinese Medicine, Changsha, Hunan, China

**Keywords:** ischemic stroke, circadian rhythms, angiogenesis, molecular mechanisms, signaling pathway

## Abstract

The circadian rhythm is an endogenous clock system that coordinates and optimizes various physiological and pathophysiological processes, which accord with the master and the peripheral clock. Increasing evidence indicates that endogenous circadian rhythm disruption is involved in the lesion volume and recovery of ischemic stroke. As a critical recovery mechanism in post-stroke, angiogenesis reestablishes the regional blood supply and enhances cognitive and behavioral abilities, which is mainly composed of the following processes: endothelial cell proliferation, migration, and pericyte recruitment. The available evidence revealed that the circadian governs many aspects of angiogenesis. This study reviews the mechanism by which circadian rhythms regulate the process of angiogenesis and its contribution to functional recovery in post-stroke at the aspects of the molecular level. A comprehensive understanding of the circadian clock regulating angiogenesis in post-stroke is expected to develop new strategies for the treatment of cerebral infarction.

## Introduction

Stroke is the second leading cause of death worldwide, and with an annual incidence of more than two million, it is the leading cause of death in China (Zhou et al., 2016; GBD 2017 DALYs, and HALE Collaborators, 2018). The incidence of stroke in China has dramatically increased over the last decade; moreover, stroke patients suffer long-term cognitive and behavioral deficits due to a lack of therapeutics focused on neural recovery post-stroke (Stubblefield and Lechleiter, 2019). Although the current intravenous (IV) recombinant tissue plasminogen activator (rt-PA) and endovascular mechanical thrombectomy have a major impact on the outcome of recanalization of intracranial vascular occlusions, the narrow therapeutic window and risk of hemorrhage result in less than 22% of patients with ischemic stroke using rt-PA, and the majority of patients still have some degree of disability, highlighting the need for new targets and therapies to improve neuro recovery and rehabilitation after ischemic stroke (Fraser et al., 2017; Barthels and Das, 2020; Marko et al., 2020).

Currently, several promising treatments have been developed in the preclinical stage, such as normobaric hyperoxia, free radical scavenger *α*-phenyl-butyl-tert-nitrone (*α*PBN), and the N-methyl-D-aspartic acid (NMDA) antagonist MK801, mainly by protect neural cells primarily by preventing excitotoxicity, oxidative stress, inflammation, or apoptosis in rodent models of cerebral ischemia (Deng et al., 2018). However, these experimental designs failed in large clinical trials, which may not have taken into account two main factors, one is that current treatments focus on neuroprotection rather than including supportive processes (Moskowitz, 2010;Chellappa et al., 2020), and the other is that endogenous circadian cyclicity is not considered in clinical translational studies. Promoting angiogenesis and subsequent increased cerebral blood flow have been confirmed to be supportive strategies for ischemic stroke (Kanazawa et al., 2019). The collateral circulation reestablished by angiogenesis partially determines the recovery of cerebral blood supply, regeneration of neurons, and reconstruction of synaptic connections between neural cells; all of that affect the degree of functional recovery of patients (Ergul et al., 2012; Ma et al., 2021). Emerging evidence suggests that angiogenesis in the penumbra and surrounding areas is a reparative process that correlates positively with the survival rate of ischemic stroke patients (Arenillas et al., 2007; Yin et al., 2015; Heydari et al., 2020).

More than this, endogenous circadian cyclicity determined the outcome of clinical translation, and circadian biology affects the mechanism of disease and response to therapies (Cederroth et al., 2019; Logan and McClung, 2019). In the rodent ischemic stroke model, neuroprotective strategies, including normobaric hyperoxia, αPBN, and MK801, were administered during the inactive (ZT3-9) and active (ZT15-21) phases, respectively; inactive phase administration of neuroprotective approaches is more effective and preserves more penumbra zone (Esposito et al., 2020). Environmental circadian disruption (ECD), induced by 6-h phase advances of the light cycle each week for 6 weeks, was shown to aggravate the stroke severity in the cerebral infarction rat model, exhibiting greater infarct size and a more pronounced inflammatory response (Ramsey et al., 2020).

The circadian clock, dominated by the mammalian endogenous circadian system, is a comprehensive regulatory system that controls the organism’s wake–sleep cycle, body temperature, hormone secretion, *etc*., and plays a critical role in metabolic regulation. In recent years, the effects of the circadian clock on stroke and angiogenesis have been extensively studied. In a clinical study of 55 stroke patients, the result showed that core clock gene Bmal1 methylation induced by PM2.5 exposure was negatively associated with the National Institutes of Health Stroke Scale (NIHSS) score (Cantone et al., 2020). More than this, Bmal1 contributes to revascularization after ischemic injury, Bmal1^−/−^ mice displayed an impaired angiogenesis ability after ischemic injury, anti-CD31-positive capillary density and VEGF protein levels arereduced in Bmal1^−/−^ mice (Xu et al., 2021). Even so, the exact mechanism of the circadian in angiogenesis after ischemic stroke remains unclear. This review focusses on introducing the potential mechanisms underlying the role of circadian rhythms in angiogenesis, and vascular remodeling and neurological outcome post-stroke, to provide a reference for investigations in related fields.

## Composition of the circadian clock in mammals

**FIGURE 1:**
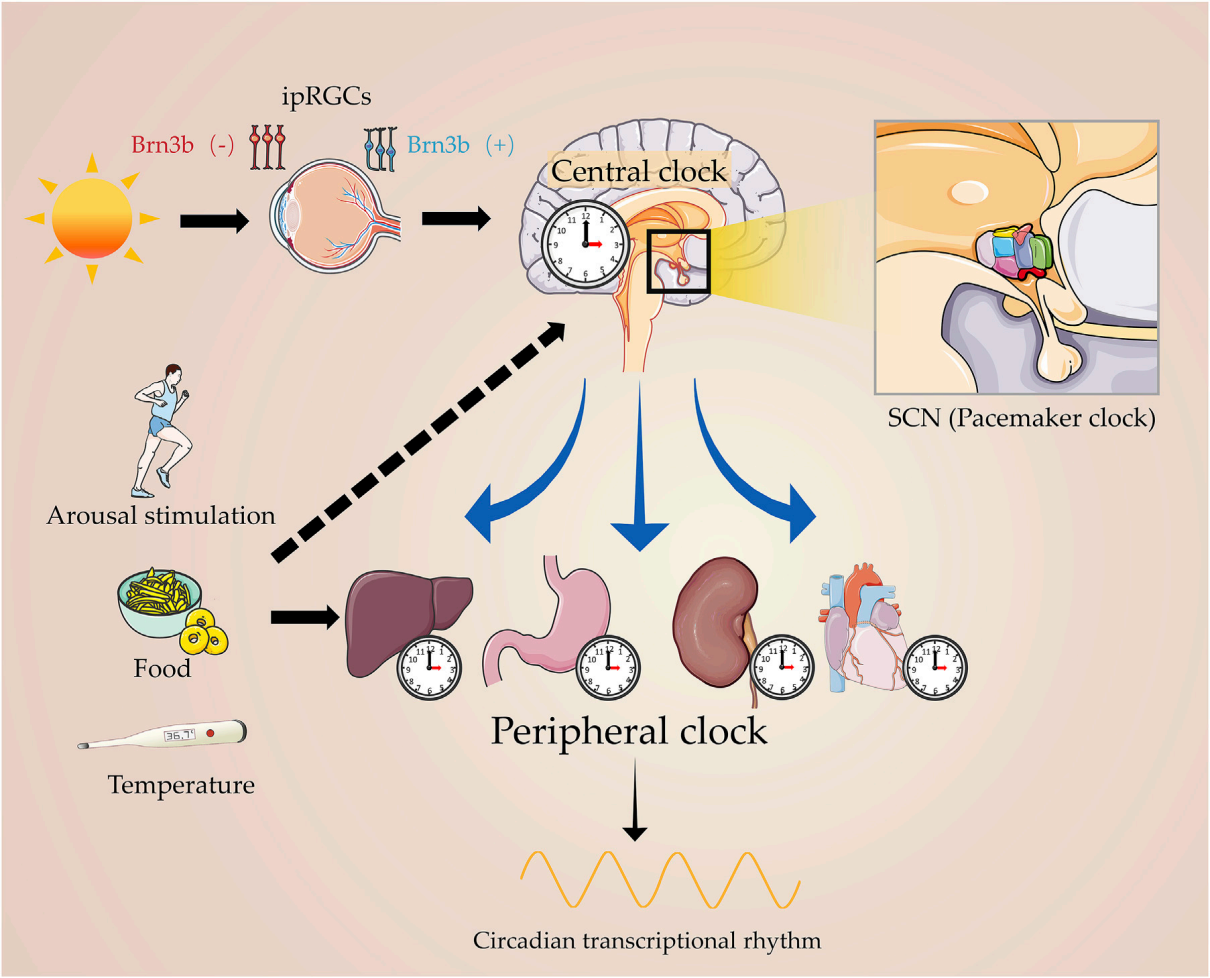
Master circadian clock and peripheral clock. Light signals the master clock, by stimulating the circadian pacemaker, SCN, which governs the peripheral clock. On the other hand, arousal stimulation coordinates with the central clock in regulating the physiological processes of human beings.

The rotation of the Earth around the axis creates an inherent dynamic ecosystem characterized by circadian rhythmic changes according to a periodic light–dark cycle of approximately 24 h. Although light provides the energy required for photosynthesis, the light–dark cycle radiation and temperature oscillations exerted by light impose a considerable amount of evolutionary pressure on photosensitive species (Rosbash, 2009), resulting in a large number of species completing life activities according to the circadian rhythm. Called “rhythms around (Circa-) a day (-Diem)” in Latin, this phenomenon is evidenced by the sleep–wake cycles of mammals, hormone levels, body temperature, heartbeat, and blood pressure (Honma, 2013; Raghow, 2018; Foster, 2020; Rhoads et al., 2020; Figure 1).

Konopka and Benzer (1971) discovered the genetic basis of rhythmic motor activity in *Drosophila*. The first mammalian circadian gene, PERIOD, was cloned in 1984, by Jeffrey Hall, Michael Rosbash, and Michael Young (Bargiello et al., 1984; Bargiello and Young, 1984; Reddy et al., 1984; Zehring et al., 1984), for which they were awarded the Nobel Prize in 2017. Subsequently, research on the molecular mechanism of mammalian circadian rhythms has revealed many additional genes that belong to the clock core loop (van der Horst et al., 1999; Bae et al., 2001), which promotes the study of circadian rhythms in the behavioral, physiological, anatomical, and molecular levels.

Circadian rhythms are influenced by photoperiod, and regulate the complicated pathophysiological processes through positive and negative feedback loops, which in turn are controlled by the central pacemaker in the suprachiasmatic nucleus (SCN) (Walker et al., 2020). The SCN receives nerve impulses conducted by intrinsically photosensitive retinal ganglion cells (ipRGCs) through the monosynaptic pathway when exposed to light on the activable spectrum (Bedrosian and Nelson, 2017). Interlocking transcription–translation feedback loops (TTFLs) are the foundation of the molecular circadian clock in mammals, which take approximately 24 h to be completed. A set of interlocked core clock genes and their protein products were involved in circadian biology *via* TTFLs, including Brain and muscle aryl hydrocarbon receptor nuclear translocator-like protein 1 (Bmal1, also known as Arntl), Circadian locomotor output cycle kaput (Clock), Cryptochrome family (Cry1 and Cry2), and Period family (Per1, Per2, and Per3) (Takahashi, 2017; Begemann et al., 2020).

Clock and Bmal1 heterodimerize transcriptional activator complexes to rhythmically activate downstream target genes containing e/e- box elements in their promoter and/or enhancer regions (De Nobrega and Lyons, 2020). The first main loop includes members of the mammalian Per and Cry families. At the early stages of the cycle, three Per and two Cry heterodimerize form a large nuclear complex in the cytoplasm (Brown et al., 2005), translocate to the nucleus upon phosphorylation by casein kinase I (CKI ε/δ) (Lee et al., 2011; Aryal et al., 2017), combined with the CLOCK-BMAL1 heterodimers at the promoter regions of Per and Cry (Duong et al., 2011), and form quaternary complexes that block the transcriptional processes of Bmal1-target genes, including their own (Kim and Lazar, 2020). Then, the Per and Cry protein levels in the nucleus decrease, the transcriptional inhibition of CLOCK-BMAL1 is relieved, and CLOCK-BMAL1 mediated transcription resume, thus allowing a new circadian cycle to begin (Cox and Takahashi, 2019).

In addition, the Per and Cry genes, the nuclear receptor (NR) Reverbs together with retinoid-related orphan receptor (ROR) subfamily forms a second loop that ensures the rhythmic expression of Bmal1 (Patke et al., 2020). Both classes of receptors have been confirmed to regulate the Bmal1 expression (Burris, 2008): RORα/β activates the transcription of Bmal1 (Sato et al., 2004), whereas Rev-erbα/β suppresses its transcription (Preitner et al., 2002; Guillaumond et al., 2005; Wang et al., 2020). Reverbα/β and RORα/β/γ competitively bind to Reverb–ROR response elements in the promoter and enhancer regions of target genes, including Bmal1, and inhibit or activate their transcription, respectively (Ueda et al., 2002). The third loop consists of the proline and amino acid-rich leucine zipper (PAR-bZIP), DBP (D-binding protein), TEF (thyrotroph embryonic factor), HLF (hepatic leukemic factor), and NFIL3/E4BP4 (nuclear factor, interleukin-3 regulated, E4 promoter binding factor 4) (Drolet et al., 1991; Fonjallaz et al., 1996; Takahashi, 2017; Kim and Lazar, 2020). Clock-Bmal1 heterodimerizes to regulate Reverbα/β, which rhythmically represses the transcription of NFIL3. NFIL3 in turn represses PAR-bZIP and DBP to regulate the rhythm of the ROR nuclear receptors (Mitsui et al., 2001; Gachon et al., 2004). The remaining factors can recognize and compete for D-box motifs on promoters and enhancers, thus activating transcription in a redundant manner (Fonjallaz et al., 1996; Gachon et al., 2004, 2006). Therefore, this cascade coordinates the interaction between the positive and negative regulation of RORE and Reverb, and shapes the oscillating expression of Bmal1 (Zhang and Heaney, 2020). An increasing body of evidence has demonstrated that circadian rhythms play pivotal roles in diverse physiological and pathological processes, including the cardiovascular system, energy metabolism, immunity, hormone secretion, and reproduction (Panda, 2016; Pilorz et al., 2018; Krueger et al., 2020).

## Circadian system and stroke

### Circadian system disruption and desynchronization contribute to stroke

The circadian system is involved in the physiological and pathophysiological processes of the human body. It is now known that the incidence of myocardial infarction and ischemic stroke occurs significantly more often in the morning, while respiratory and other inflammatory diseases tend to become exacerbated at night (Paschos and FitzGerald, 2010; Tsimakouridze et al., 2015). The influence of circadian rhythms on stroke is first manifested in the difference in stroke onset time: the incidence of stroke increases significantly between 6 a.m. and 12 p.m., and the frequency of onset of hemispheric stroke was significantly (*p* = 0.0001) higher between 6:01 a.m. and 12:00 p.m. (56.1%) than between 12:01 and 6 p.m. (20.2%), 6:01 p.m. and 12:00 a.m. (8.2%), and 12:01 and 6 a.m. (15.5%) (Chaturvedi et al., 1999; Ripamonti et al., 2017; Fodor et al., 2021). With the deepening of research, the environmental circadian disruption (ECD) model, induced by 6-h phase advances of the light cycle each week for 6 weeks, is widely used in the research of circadian rhythms (Sellix et al., 2012; Hill et al., 2021). Circadian clock dislocation induced by ECD has confirmed increased stroke severity in rats with cerebral infarction; ECD intervention group showed greater infarct size and a more pronounced inflammatory response (Ramsey et al., 2020). Circadian disruption induced by a genetic mutation also caused severe cardiovascular dysfunction (Chellappa et al., 2019), and mutations of core clock genes (i.e., Bmal1, Clock, and Npas2) could decrease mean blood pressure and disrupt sympathoadrenal responses (norepinephrine and epinephrine) to stress, which contribute to the incidence of stroke (Curtis et al., 2007).

The core clock genes widely distributed across the brain affect most fundamental physiological processes, including the trigger factors of stroke, such as arterial blood pressure, heart rate, coagulation balance, and other rhythmic events (Li et al., 2013; Fodor et al., 2021). In recent years, with the increasing application of ambulatory blood pressure monitoring (ABPM) in stroke research, more and more evidence indicates that circadian blood pressure is closely related to the incidence of stroke, an especially circadian rhythm disturbance of blood pressure as an independent risk factor for stroke (Pierdomenico et al., 2016; Cai et al., 2017). Such phenotypes referred to as “non-dipper” (failure to downregulate BP in the nocturnal phase) or “super-dipper” (exacerbated hypotension during the nocturnal phase) are both known as the individual risks of stroke morbidity and mortality *via* the hemodynamic mechanism and cerebral hypoperfusion (Smolensky et al., 2007; Verdecchia et al., 2012). Circadian clock gene mutant mice were used in further study; deletion of Bmal1 leads to the super-dipper phenotype in the nocturnal phase, by impairing the transcriptional level of angiotensinogen (Agt), which plays a critical role in vasoconstriction (Chang et al., 2018).

In addition, diurnal changes were also observed in coagulation and the fibrinolytic systems, which are closely related to morbidity and severity degree of ischemic stroke (Wang et al., 2018). During the morning hours of the high incidence of stroke, due to the effects of circadian rhythms, the human body is in a prothrombotic state of hypo-fibrinolysis and hyper-coagulation (West et al., 2021). Furthermore, the loss of circadian fluctuations in euglobulin clot lysis time (ELT) was observed in Clock mutant and Cry1/Cry2 double knockout (Cry1/2-deficient) mice (Ohkura et al., 2006). ELT is inversely proportional to fibrinolytic activity, with ELT in wild-type mice showing a circadian change in ELT that peaks at 21:00 (inactive phase). The Clock and Cry mutant mice showed diametrically opposite results: the ELT of the Clock mutant was consistently decreased, whereas the ELT of the Cry1/2-deficient mice increased significantly and did not differ between 9:00 and 21:00 (inactive phase and active phase) (Ohkura et al., 2006). A continuous reduction of clot lysis time of euglobulin was reported in Clock deficient mice, while a significantly increased level was detected in Cry1/Cry2 mutant mice, following the loss of circadian rhythm. Bmal1 deficiency, manifested in a hypercoagulable state, increases arterial and venous thrombosis, resulting in endothelial dysfunction (Hemmeryckx et al., 2019). In addition, the disruption of circadian rhythms in the liver and plasma plasminogen activator inhibitor-1 (PAI-1) was observed in Bmal1^−/−^ mice, as no significant difference was observed in the expression of factor (F)VII, protein S, and anti-thrombin, and PAI-1 at both zeitgeber time (ZT)2 and ZT14 (Hemmeryckx et al., 2019). Global Bmal1 knockout mice showed larger areas of pathological remodeling and thrombosis in chronically reduced blood flow than WT mice (Anea et al., 2009). These findings suggest that circadian biology influences blood pressure and coagulation balance; once the circadian system is dislocated, the rhythm fluctuations of blood and coagulation disappear, which become a potential factor for the incidence of stroke.

### Stroke induces circadian disruption

As previously described, internal circadian dysregulation may contribute to the incidence of stroke; conversely, the stroke itself can lead to the desynchronization of endogenous circadian rhythms by directly affecting the SCN, or by disrupting the clock mechanisms of neurons, glia, and endothelial cells (Kostenko and Petrova, 2018; Fodor et al., 2021). Clinically, strokes are positively related to anxiety-depressive and affective disorders, and are accompanied by a high frequency of sleep disorders, and desynchronization in daily curves of the heart rate (HR) and blood pressure (BP) (Jain et al., 2004; Hidehiro et al., 2007; Kostenko and Petrova, 2018). A 2004 study found that diurnal blood pressure changes were canceled in most acute stroke patients, whose blood pressure typically dropped by at least 10% at night (Jain et al., 2004). In addition, ischemia injury produces a phase advance of Per1 expression (Karmarkar and Tischkau, 2013) and alters the rhythm of melatonin secretion in the pineal gland, thereby regulating the expression of Bmal1, both of which are critical for cell survival in neuronal ischemia (Eckle et al., 2012; Beker et al., 2019).

Taken together, these results suggest that the circadian system is involved in vascular function and regulates the expression of key factors in the hemostatic and fibrinolytic system, leading to an increased risk of non-dipper blood pressure and prethrombotic phenotype, thereby increasing the risk of stroke events. Intriguingly, the occurrence of cerebral infarction promotes the desynchronization of endogenous biorhythms and disrupts the expression pattern of clock genes, suggesting that intervention of the circadian system, such as environmental modifications, chronotherapy, and targeting the clock genes may be a potential target for treatment after cerebral infarction.

## Angiogenesis and stroke

### Angiogenesis is critical for the recovery process of ischemic stroke

The cerebral blood flow decreases below the critical level after cerebral artery occlusion, resulting in neuronal cell death and brain infarction, which leads to neuronal electrical activity cessation and the development of functional deficit (Astrup et al., 1981; Campbell and Khatri, 2020). Thus, the re-establishment of a functional cerebral microvascular network plays a far more important role in regional blood supply and stroke recovery (Yin et al., 2015). The majority of vessels are in a resting state under normal physiological conditions and induce angiogenesis after ischemic cerebral stroke in humans (Marti et al., 2000; Hayashi et al., 2003). Angiogenesis is the formation of a new micro-vessel network that branches off from pre-existing ones (Carmeliet and Jain, 2011; Ruan et al., 2015), which supplies oxygen and nutrients to the affected areas that occur in the boundary zone (Beck and Plate, 2009). Importantly, a high degree of neovascular function facilitates neurorehabilitation and functional recovery to some extent (Slevin et al., 2006; Arenillas et al., 2007; Chen et al., 2018; Kanazawa et al., 2019). Therefore, promoting post-ischemic angiogenesis is a key conceptual target for the treatment of ischemic cerebral stroke in clinical practice.

### Angiogenesis after cerebral infarction is regulated by considerable factors

The angiogenesis process in humans occurs 3–4 days following ischemic injury and is complexly regulated by angiogenic growth factors and inhibitors that promote and induce endothelial cell migration and proliferation (Krupinski et al., 1994). Numerous pro-angiogenic factors are involved in the angiogenesis process, including vascular endothelial growth factor (VEGF) (Issa et al., 1999; Sun et al., 2003; Chan et al., 2020), basic fibroblast growth factor (bFGF) (Issa et al., 2005; Kigel et al., 2011), platelet-derived growth factor (PDGF) (Renner et al., 2003; Clarke, 2020), transforming growth factor-beta (TGFβ) (Mahmoud et al., 2011; Jarad et al., 2017), matrix metalloproteinases (MMPs) (Sounni et al., 2011; Lv et al., 2018), and thrombospondin-1 (TSP-1) (Lin et al., 2003); the abovementioned related genes are activated after ischemic stroke, thereby promoting the process of angiogenesis (Yin et al., 2015).

**FIGURE 2:**
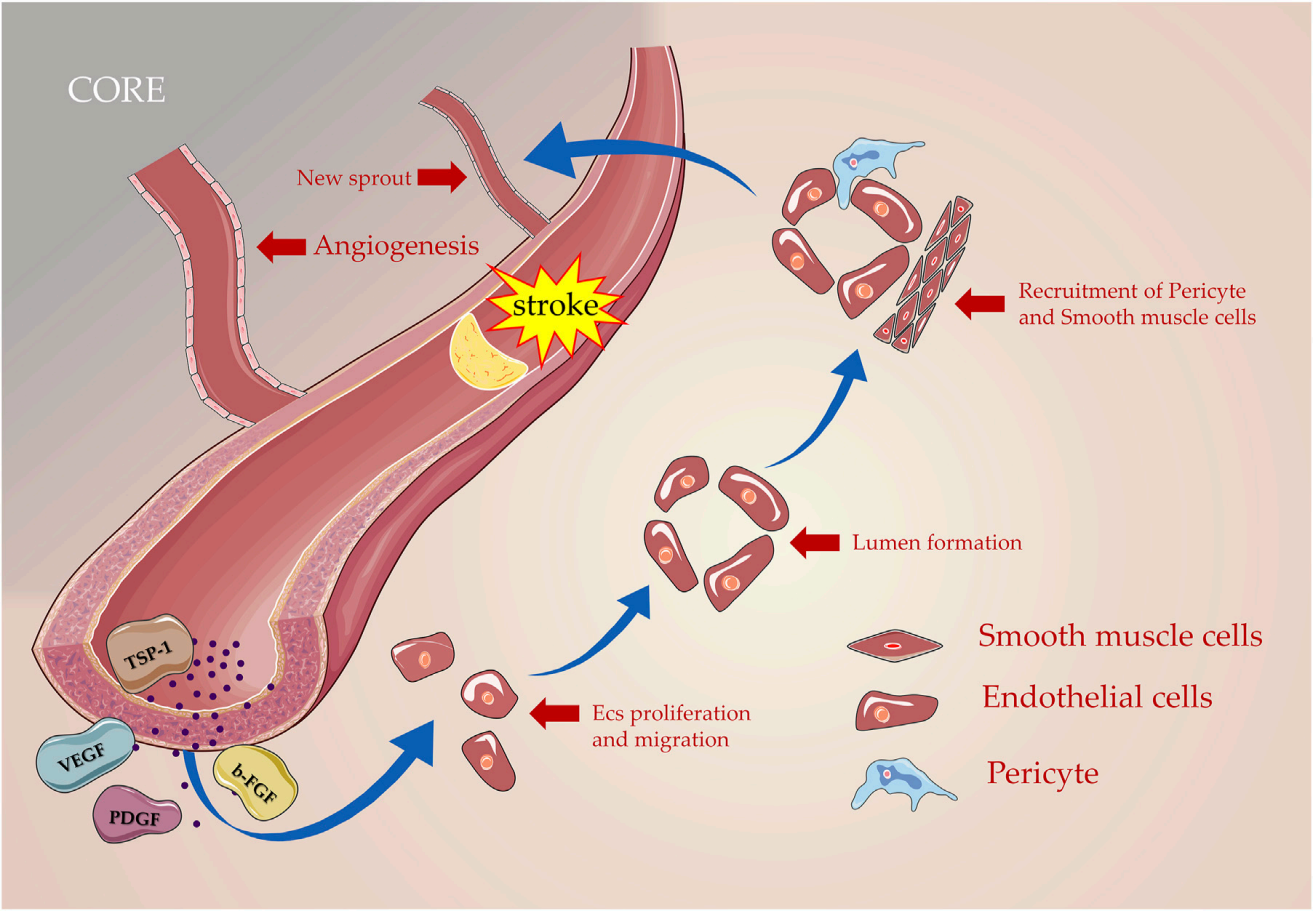
Processes of angiogenesis in post-stroke. Numerous angiogenic inducer factors including VEGF, PDGF, and b-FGF are involved in this process as proliferation, migration, lumen formation, and recruitment of pericyte; new blood vessels are formed gradually based on the original blood vessels.

When it comes to suffering from ischemic and hypoxia injury, ECs were activated, and a large number of MMPs are released to lyse the extracellular matrix (ECM), basement membrane, and matrix so that pericytes are detached from the vessel wall and basement membrane (Yang and Rosenberg, 2011). Vascular permeability increases to cope with the exudation of VEGF, thereby the plasma proteins assist ECs to migrate by forming the configuration of the primitive scaffold (Hong et al., 2019). Under the synergistic action of the aforementioned growth-promoting factors, ECs proliferate and migrate, and activated ECs aggregate and shape the lumen by migrating to a distinct section to induce neovascularization formation (Jain and Carmeliet, 2012). Furthermore, adhesion molecules were secreted to pull the sprouting blood vessel forward organizing it into a network of new blood vessels (Marti et al., 2000). The new capillaries rely on the coordinated remodeling of the basal matrix and the extension of the ECs behind the terminal cells (Li and Carmeliet, 2018), including the recruitment of pericytes and smooth muscle cells, which grow linearly on the original blood vessels in the form of sprouting (Chiaverina et al., 2019; Figure 2).

Angiogenesis after cerebral infarction is a tightly regulated and complex process that involves many networked pathways. Immune cells can secrete a variety of cytokines through a variety of inflammation-related signaling pathways to affect the proliferation, migration, and differentiation of endothelial cells, thereby participating in the process of angiogenesis (Zhu et al., 2021). Brain-resident microglia are first activated within an hour of ischemic stroke (Planas, 2018): M1-type microglia secrete pro-inflammatory factors, including interleukin-1 (IL-1), IL-6, tumor necrosis factor α (TNF-α), and MMP-9, while M2-type microglia produce IL-10, transforming growth factor β (TGF-β), insulin-like growth factor, and VEGF, which are synergistically involved in the process of angiogenesis (Dabrowska et al., 2019). In addition, approximately 24 h after stroke, immune cells in the peripheral blood are recruited into the damaged brain, including macrophages, natural killer (NK) cells, neutrophils, and T lymphocytes that migrate to the damaged area and interact with components of the blood–brain barrier (Duris et al., 2018; Planas, 2018; Chen et al., 2019). Previous studies have shown that the increase in the number of macrophages can significantly promote angiogenesis after stroke (Manoonkitiwongsa et al., 2001), and T helper 17 cells promote angiogenesis and increase cerebral blood flow in the ischemic penumbra by partially promoting EC migration and sprouting; cellular infiltration of Th2 increases IL-4, IL-10, and TGF-β, and decreases IFN-γ expression, which are key factors in promoting angiogenesis during stroke recovery (Kwee et al., 2018; Zhu et al., 2021).

## Circadian system in angiogenesis

**FIGURE 3:**
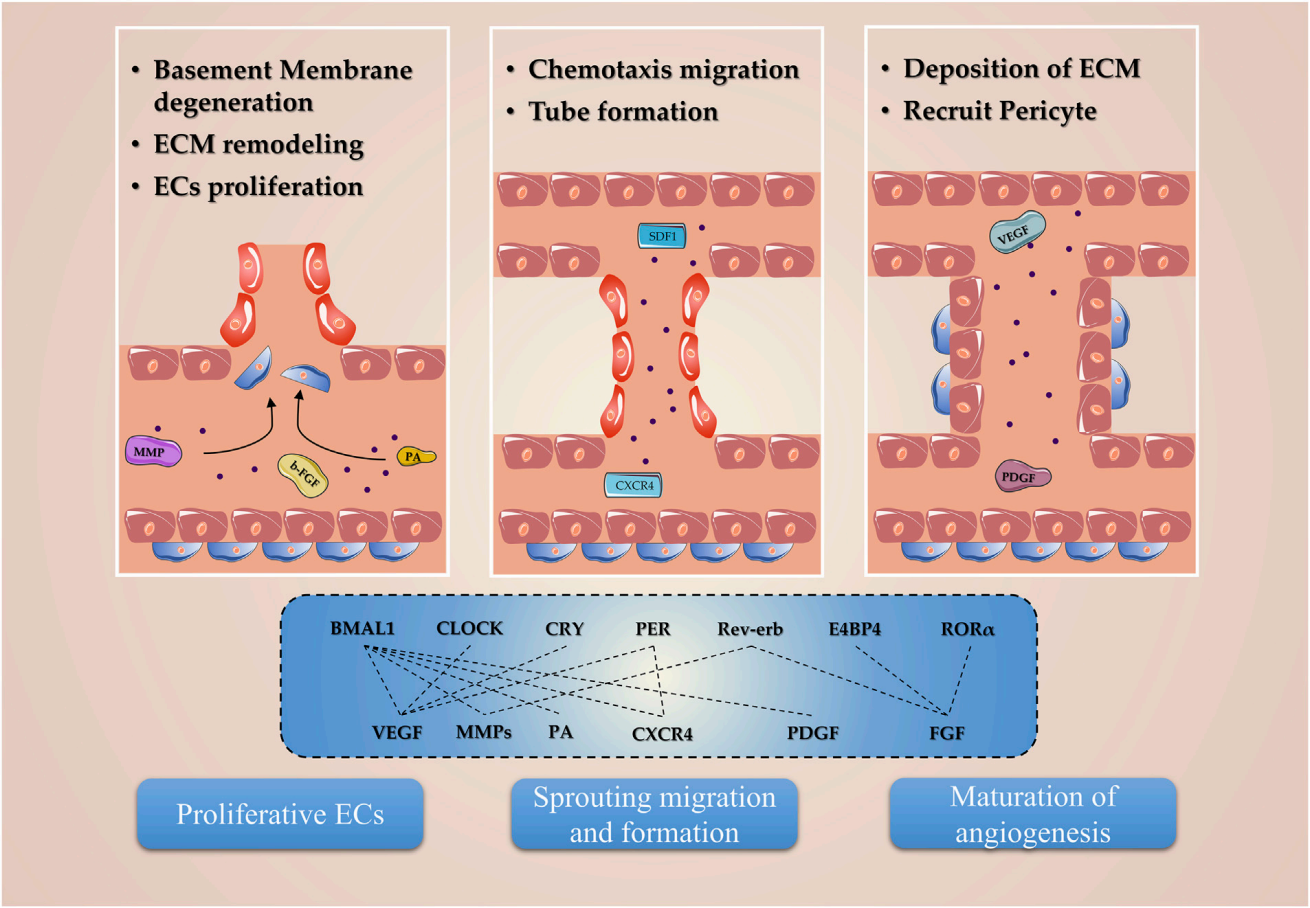
Molecular mechanism of circadian clocks involved in angiogenesis. The circadian clocks, including Bmal1, Clock, Cry, Per, Reverb, E4BP4, and RORα, regulate the expression of the angiogenic factor and are involved in the proliferation, migration, and tube formation phenotypes in endothelial and pericyte.

Cells closely related to angiogenesis, whether endothelial cells or pericytes, have been shown to have internal clocks and are inextricably linked to angiogenesis after ischemic stroke (Takeda et al., 2007; Mastrullo et al., 2022). The circadian clocks govern this event by regulating various ECs and pericyte phenotypic processes, including oscillation parameters of angiogenic regulatory factors, basement membrane (BM) degradation, EC migration and proliferation, and pericyte recruitment (Figure 3).

### Circadian clocks are involved in the ECM and BM degeneration

The BM is an amorphous structure located near the lumen of endothelial cells or at the basal side of epithelial cells, which is composed of ECM, a complex structural entity surrounding which containing three major molecular levels: 1) structural proteins, collagen, and elastin; 2) specialized proteins, for example, fibrillin, fibronectin, and laminin; and 3) proteoglycans (Adibhatla and Hatcher, 2008). Previous studies revealed that BM degeneration and remodeling of ECM contribute to angiogenesis in post-stroke, which are key responses to detach pericytes from the vessel wall and allow ECs to migrate and invade the surrounding tissue (Liu et al., 2014). The proteases, such as the MMP family, are critical for this cascade centered on BM degeneration and ECM remodeling, which allows the pericytes to detach from undergoing angiogenesis vessels (Jabłońska-Trypuć et al., 2016). At the same time, ECs release ECM combined angiogenesis growth factors, exposing potential proangiogenic integrin-binding sites in the ECM, generating promigratory ECM component fragments, and collectively contributing to angiogenesis (Rundhaug, 2005).

Given that MMP2 and MMP9 are expressed in SCN, and MMP9 mRNA decreased under circadian disruption treatment, circadian rhythms should be taken into account in the expression and ECM degeneration of MMPs (Taishi et al., 2001; Schloss et al., 2016). More than this, MMP2 and MMP9 are confirmed to be elevated in Bmal1^−/−^ mice in remodeled arteries, which further contribute to ECM and BM degeneration (Anea et al., 2010). But the expression of MMP9 mRNA and protein uniformly increased under the synthetic Rev-erb agonists, and SR9009 intervention during the acute ischemic injury phase (Stujanna et al., 2017). Moreover, chronic circadian rhythm disruption alters matrix degradation by increasing MMP3 and MMP13, and inhibition of Bmal1 upregulates the mRNA and protein of MMP3 and MMP13 (Song et al., 2021).

The PA (plasminogen activator), including uPA (urokinase plasminogen activator) and tPA (tissue plasminogen activator), also plays a critical role in the procession of BM degeneration and ECM remodeling (Zhai et al., 2022). uPAR (urokinase-type plasminogen activator receptor) binds with pro-uPA (zymogen form) to trigger the proteolytic cascade, which leads to the degeneration of ECM (Stephens et al., 1989). In addition, uPA and tPA, respectively, activate the plasminogen, and target downstream MMP2, MMP3, and MMP9 to dissolve fibrin and mediate intercellular infiltration, which contributes to the ECM remodeling and further enhances the angiogenic ability (Hahn-Dantona et al., 1999; Pepper, 2001; Song et al., 2016; Su et al., 2016). Both tPA and uPA are expressed in SCN, and the protein of tPA exhibited a rhythmic pattern with a peak during the night in mice (Cooper et al., 2017, 2018). In contrast to MMP9, 8 h of sleep deprivation followed by 2 h of recovery increased tPA mRNA expression in SCN, suggesting that tPA is involved in modulating the circadian phase shift (Taishi et al., 2001). Intriguingly, the expression of tPA exhibits a BmalL1-dependent manner, and an increased mRNA level of tPA was observed in Bmal1^−/−^ mice brain tissue regardless of diurnal time (Hemmeryckx et al., 2019). Taken together, these observations may have implications if it is feasible to regulate the effect of ECM degeneration on angiogenesis after cerebral infarction by modulating the rhythmic expression of the circadian clock.

### Circadian clocks regulate proangiogenic regulatory factors

**FIGURE 4:**
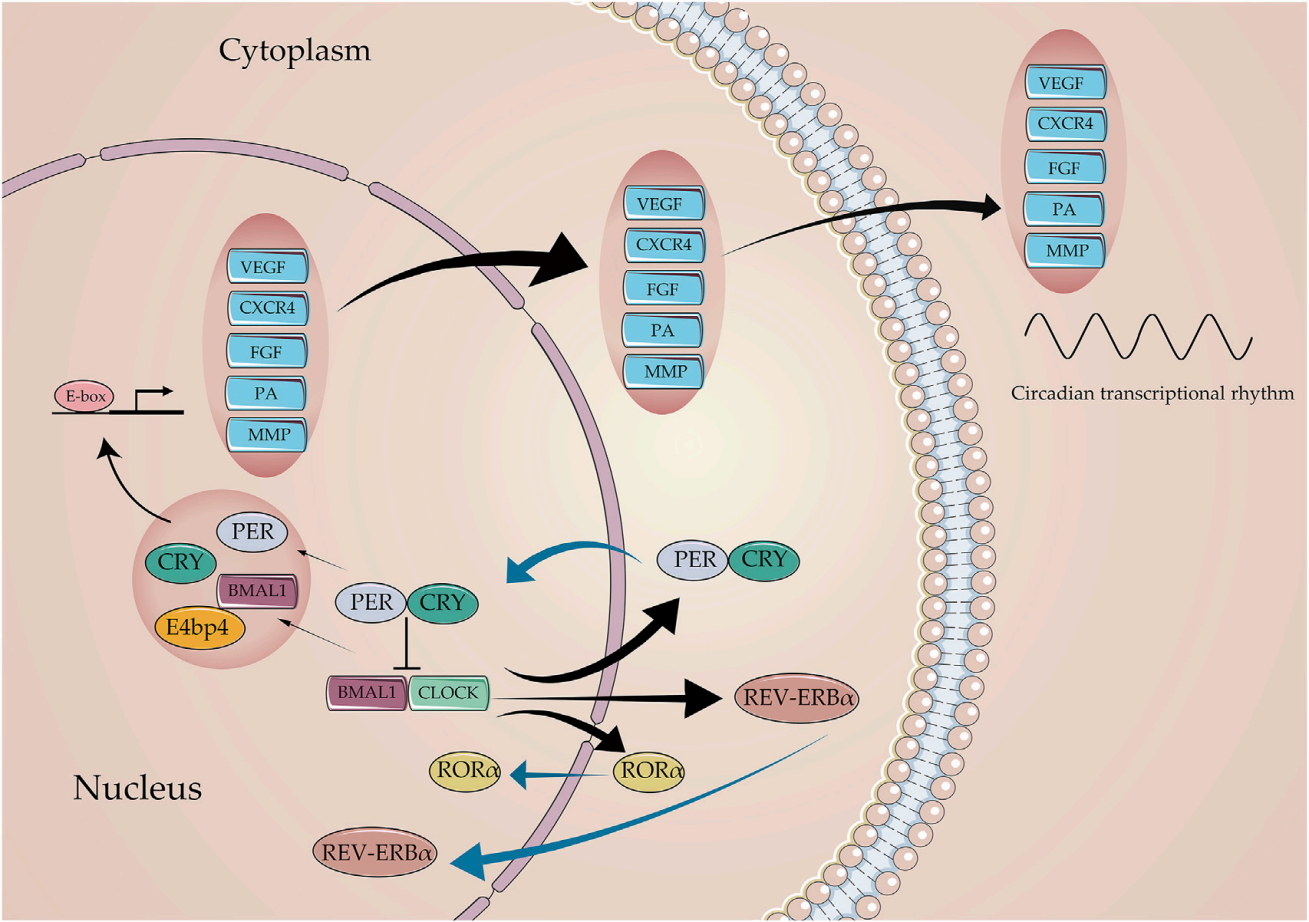
Core circadian clock genes regulate the expression of angiogenic factors. The Bmal1: Clock initiates the transcriptional process of the Per, Cry, Rev-erb, and ROR families. In return, Per and Cry suppress the transcriptional process of Bmal1, Clock through the E-box. Critical angiogenic factors, including VEGF, CXCR4, FGF, PA, and MMP, are regulated by circadian clocks to varying degrees in molecular mechanisms.

The number of angiogenic regulatory factors in the blood determines the functions of endothelial and pericyte, acting as the key parameters for the angiogenesis process of ischemic stroke. Generally, VEGF is a well-known angiogenic mediator in stroke that initiates different steps in the angiogenic cascade, such as endothelial cell proliferation, migration, and differentiation (Geiseler and Morland, 2018). As known downstream targets of the circadian clock network, VEGF protein displays the oscillation expression pattern, which is regulated by the core circadian component Bmal1, Clock, Per, and Cry genes (Koyanagi et al., 2003). The overexpression of Bmal1 promotes the luciferase activity of VEGF; however, knocking down the VEGF expression reversed the promoting effects of Bmal1 in pro-angiogenesis in HUVECs Xu et al., (2021). The human VEGF promoter activity follows a 24-h circadian pattern under the 12 h light–dark (LD) cycle, and the core circadian gene Bmal1 plays a transcriptional role by targeting the region of the VEGF promoter (Jensen et al., 2012). In addition, the fluctuation of the serum VEGF level displayed a rhythmic pattern peaking during noon, while ECD induced by jet lag impaired this fluctuation by decreasing the VEGF serum level (Tsuzuki et al., 2021). Moreover, Per2 and Cry1 act as critical rhythmic regulators of the hypoxia-induced expression of the VEGF; these observations indicate the existence of a negative feedback loop that rhythmically suppresses the transcriptional upregulation of VEGF under hypoxic conditions, resulting in the circadian fluctuation of VEGF expression and angiogenesis (Koyanagi et al., 2003; Figure 4).

The FGF family and their receptors are known to govern angiogenic functions in post-stroke, by activating FGF receptors on endothelial cells, inducing the release of angiogenic factors from other cell types, promoting the proliferation and migration of ECs, and enhancing the biological activity of VEGF (Beenken and Mohammadi, 2009). FGF21, the subtype of the FGF family, has been confirmed to promote angiogenesis and endothelial progenitor cell function when suffering ischemic injury (Dai et al., 2021). Many research studies showed that circadian clocks are involved in the regulation of FGF21, due to the mRNA and protein of FGF21 displaying a circadian oscillation in both rodents and humans (Oishi et al., 2008; Yu et al., 2011; Foo et al., 2013). Furthermore, research determined that the up-regulation of FGF21 by berberine is abided by the Bmal1-dependent mechanism, and the knockdown of Bmal1 abolished the increased expression of FGF21 in response to berberine (Hirai et al., 2019). In the human cell lines, FGF21 displayed a circadian rhythm expression pattern; however, its mRNA altered when the major component of circadian clocks, E4BP4 (E4-binding protein 4), was knockdown. E4BP4 directly binds the region of the FGF21 promoter, thereby playing the transcriptional suppressor role (Tong et al., 2010). More than this, other two kinds of circadian components, RORα and Rev-erbα, have also been found to directly regulate the expression of FGF21 (Estall et al., 2009; Wang et al., 2010). These research studies have implicated that the rhythmicity of the VEGF and FGF are directly controlled by the circadian clocks. Enhancing the angiogenic regulatory factor circadian oscillation by regulating the circadian clock’s gene expression may be a novel treatment for angiogenesis in ischemic stroke.

### Circadian clock components affect the phenotypes of endothelial pericytes

The chemotaxis, migration, and proliferation phenotypes of ECs play a promoting role in angiogenesis under the participation of various growth factors and chemokines. The chemokines, a family of small proteins, are represented by SDF1 (stromal cell-derived factor-1, also known as cysteine-X-cysteine chemokine ligand 12, CXCL12), which is a critical regulator for vascular morphogenesis and angiogenesis by inducing chemotactic migration and the invasive response of ECs, stimulating ECs to become motile and protrude filopodia, forming the tip cells (Salvucci et al., 2002; Jakobsson et al., 2010; Carmeliet and Jain, 2011). Intriguingly, SDF1 and receptor CXCR4 (cysteine-X-cysteine chemokine receptor 4) have also been demonstrated to exhibit rhythmic expression patterns, by detecting the oscillating expression in mice, *Pelteobagrus vachellii*, and human organisms (Scheiermann et al., 2012; Spinosa et al., 2017; Qin et al., 2021). While this pattern is circadian clock-dependent, fluctuations of CXCR4 at ZT5 and ZT13 disappeared in *Bmal1* ^*−/−*^ mice housed for 1 week in the 12 h light–dark (LD) cycle (Lucas et al., 2008). Moreover, the expression of CXCR4 was decreased in *Per2* ^*−/−*^ mice, which may be the potential mechanism of the circadian clock involved in the process of angiogenesis (Sun et al., 2014).

As for other phenotypes of ECs, the circadian clock genes in ECs, such as *Bmal1*, and *Per2*, are correlated with the angiogenic ability of ECs, such as migration, and proliferation. In HUVECs, overexpression of Bmal1 promotes angiogenic activity, including proliferation, migration, and tube formation ability. Furthermore, *in vitro* research, Bmal1^−/−^ mice impaired angiogenesis by labeling CD31 and measuring the decreased VEGF protein level after peripheral ischemic injury (Xu et al., 2021). Similarly, endothelial isolated from *Per2* ^*−/−*^ mice substantially lost the vascular networks and proliferation ability, further contributing to increasing senescence (Wang et al., 2008). Following research explored the impact of *Per2* in EPCs (endothelial progenitor cells); *Per2* ^*−/−*^ EPCs displayed impaired proliferation, migration, tube formation, and adhesion (Sun et al., 2014). Jet lag plays an antiangiogenic role and inhibits blood reperfusion after ischemic injury; further loss-of-function studies explore the relation between *Cry* and HUVEC (human umbilical vein endothelial cell) phenotypes, which confirmed that knockdown of *Cry1* and *Cry2* in HUVECs inhibited proliferation, migration, and tubular morphological features (Tsuzuki et al., 2021).

Pericytes are involved in the above three phases of angiogenesis, including 1) BM degeneration and ECM remodeling, 2) sprouting migration and formation, and 3) maturation and termination of angiogenesis (Armulik et al., 2011). For blood vessels to function properly, neovascular must recruit mature pericytes and be covered with parietal cells. Then, pericytes attach to EC and regulate the deposition of extracellular matrix and the formation of endothelial tight junction proteins, contributing to the stabilization of the new lumen. Progressive loss of pericyte was observed in Bmal1^−/−^ mice, which contributes to the blood–brain barrier hyperpermeability and weakened angiogenesis (Nakazato et al., 2017). Recent studies further explored the potential mechanism of circadian clocks in pericytes. First, human primary pericytes indeed display a rhythmic activity and induced the circadian rhythmicity of HUVECs in the EC–pericyte contact co-culture environment. Also, core circadian gene Bmal1 was confirmed to involve in the maturation and morphology of the vascular structure (Mastrullo et al., 2022).

The pericyte marker, PDGF-β (platelet-derived growth factor), is also worth mentioning. To stabilize endothelial channels, a large amount of PDGF-B is released, which is essential for physiologic angiogenesis through a moderate VEGF dose (Hellberg et al., 2010). Its co-delivery normalizes high VEGF angiogenesis and expands the therapeutic window of PDGF (Banfi et al., 2012). In the maturation and termination phase of angiogenesis, the survival of endothelial relies on the VEGF secretion of pericytes; thus, the close interaction between pericyte and endothelial protects endothelial cells from VEGF withdrawal and confers resistance to VEGF blockade (Sennino et al., 2009). PDGF-B regulates pericyte proliferation and migration, and induces their recruitment to the nascent sprout by binding to the PDGF receptor β (Dubrac et al., 2018; Imhof et al., 2020). PDGF and circadian genes interact with each other, and jointly regulate cell proliferation. PDGF-BB increased Bmal1 protein and mRNA expression in a time-dependent manner while transiently up-regulating Clock, Per1, Per2, Cry1, and Cry2 mRNA expression, in turn; Bmal1 is involved in the regulation of PDGF-BB, and the knockdown of Bmal1 impaired the proliferation induced by PDGF-BB (Takaguri et al., 2020).

### Circadian participates in angiogenesis by regulating the immune response

The acute immune response of stroke is a characteristic of cerebral ischemia and is mediated by a large number of immune cells, chemokines, growth factors, hormones, and cytokines (Iadecola et al., 2020). As aforementioned, the M1 and M2 polarized phenotypes of microglia/macrophages participated in the brain damage and repair; the polarization of microglia to the M2 phenotype increases angiogenesis in the ischemic penumbra, and reduces infarct volume and neural validation (Jetten et al., 2014; Liu et al., 2018; Shang et al., 2020). Far beyond these, a large number of immune response-inducing cytokines are closely related to angiogenesis, including VEGF, MMP, ILs, PDGF, and monocyte chemotactic protein-1 (Mcp-1/Ccl2) involved in immune response-mediated angiogenesis (la Sala et al., 2012; Zhu et al., 2021).

Circadian governs the immune response. First, the expression of immune mediators follows a circadian oscillatory pattern, and the number of immune cells peaks in the circulation during the resting phase (nighttime in humans and daytime in rodents) and decreases during the active phase, whereas levels of related cytokines peak at the onset of active phase (Haus and Smolensky, 1999; Scheiermann et al., 2013). In addition, circadian clock proteins regulate the immune mediators. The genes of intrinsic circadian clocks, such as Bmal1, can regulate IL-6 production by modulating the transactivation process (Nakazato et al., 2017). The activation of the nuclear receptor Rev-erbα can reduce the M1 polarization of macrophages induced by inflammation (Cui et al., 2021). Moreover, Rev-erbα and Per1 have been shown to mediate the inflammatory response of chemokine and cytokines (Gibbs et al., 2012); significantly increased expression of Ccl2 and IL-6 was observed in glial cells with Per1 knockdown, and Ccl2 is elevated in Rev-erbα knockout mice (Sugimoto et al., 2014). Furthermore, studies have shown that Rev-erbα directly binds the specific binding motif of the Ccl1 promoter region that suppresses the Ccl2 expression, and impairs cell adhesion and migration (Sato et al., 2014). Ccl2 affects cell adhesion and migration, and acknowledges as a proangiogenic factor (Hong et al., 2005; Zhu et al., 2021). The overexpression of Ccl2 increased the human umbilical cord-derived mesenchymal stem cell (hUC-MSC) migration ability, thereby promoting angiogenesis in the ischemic penumbra region of MCAO rat (Lee et al., 2020).

Numerous studies have repeatedly confirmed that the inflammatory response plays a multi-stage and complex role in the progression and pathogenesis of cerebral infarction. As aforementioned, pro-inflammatory cytokines can promote angiogenesis after cerebral infarction, but excessive pro-inflammatory cytokines have adverse effects. Conversely, anti-inflammatory cytokines exert protective effects after cerebral infarction, but excessive anti-inflammatory cytokines produce immunosuppressive effects. Therefore, there is an urgent need to strike a balance between pro- and anti-inflammatory signaling to improve outcomes in cerebral infarction (Zhu et al., 2021). ECD leads to the disruption of the balance between pro-inflammatory and anti-inflammatory cytokine gene expression, which may be closely related to the abnormal oscillation of immune cells and the regulation of circadian clock proteins (Ramsey et al., 2020). The regulation of various immune cells, cytokines, and immune cell subtypes by the circadian system plays a critical role in the process of angiogenesis after cerebral infarction; thus, further studies are needed to elucidate the exact mechanism by which the circadian system regulates immune responses and induces angiogenesis.

## Conclusion and perspectives

Although no circadian clock-related drugs have been reported for ischemic stroke by targeting angiogenesis in so far, collectively, these research studies provide new therapeutic insights into the angiogenesis of ischemic stroke. Currently, several strategies have been proposed to correct clock disruption and desynchronization, to restrain the negative consequences if circadian rhythm impacts are unavoidable. These applications mainly include three aspects: environmental modifications, chronotherapy, and targeting the clock genes.

Environmental modifications also contain the high intensity of light, activity, and therapy during the day and night. Previous experiments proved that ambient light influences the occurrence and development of diseases. Mice exposed to low-dose light (5 lux) at the rest phase displayed a reduction of hippocampal VEGF and BDNF levels, and were accompanied by depressive-like symptoms (Walker et al., 2020). Thus, environmental light modifications may be the potential therapy for post-stroke. In addition, some ongoing clinical trials are carried out to explore whether light therapy and blue light exposure interventions are helpful for neuroplasticity in post-stroke (NCT05247125); bright intensive care unit rooms, especially exposed to visible daylight, have been proven to reduce delirium and its complications (Oldham et al., 2016).

Given the differences between important physiological parameters affecting stroke incidence and recovery, such as blood pressure and fibrinolytic status in humans during the active and inactive phases, and the resulting differences in circadian rhythm that may affect drug response, chronotherapy is growing in popularity (Hermida et al., 2016). Clinical studies indicated that the incidence of cardiovascular and cerebrovascular diseases decreased significantly after ingesting the entire daily dose of ≥1 hypertension drug before bedtime compared to the active period (Bowles et al., 2018; Hermida et al., 2020). Similarly, intravenous thrombolysis with rt-PA between the active phase (6:00-18:00) appears to be less effective. Also, compared with the inactive phase (18:00-6:00) interval, intravenous thrombolysis was safer and had lower rates of hemorrhagic transformation in patients who started intravenous thrombolysis between noon and midnight (Cappellari et al., 2014). Other studies have also shown a circadian variation of rt-PA in patients with ischemic stroke; intravenous thrombolysis with rt-PA during the active phase can significantly improve the modified Rankin scale compared with the inactive phase (21:00-9:00) (Vilas et al., 2012).

As aforementioned, circadian clocks regulate the molecular expression and function state of angiogenesis to various extent; therefore, another therapeutic strategy may be the changes of the phase of the circadian clock, by manipulating the rhythmic phase of circadian clocks closer to the physiological state. Many synthetic compounds, such as small-molecule modifiers, that affect the phase, amplitude, and cycle of circadian rhythm have been extensively studied, and it is expected to be realized soon in clock-targeted therapy of angiogenesis after cerebral infarction (Chen et al., 2013).

The circadian rhythm and angiogenesis modulate the majority of the processes involved in mammalian physiology and pathology. Increasing evidence indicates interactions between circadian rhythm and angiogenesis; various states during post-stroke state recovery revealed direct interactions between rhythmically angiogenesis and stroke. A better comprehension of the molecular mechanisms of the interaction between the circadian rhythm and ischemic stroke angiogenesis would help accelerate the future development of stroke therapies.
